# Supplementary material for: Unlocking genome engineering in Alcaligenes faecalis by exploiting its native type I-F CRISPR-Cas
Source: Microbiol Spectr. 2026 Mar 25;14(5):e02786-25. doi: 10.1128/spectrum.02786-25 (PMC13141931; doi:10.1128/spectrum.02786-25)
Supplement: Supplemental material — Fig. S1 and S2; Tables S1 to S3. [file spectrum.02786-25-s0001.docx]

**SUPPLEMENTAL DATA**


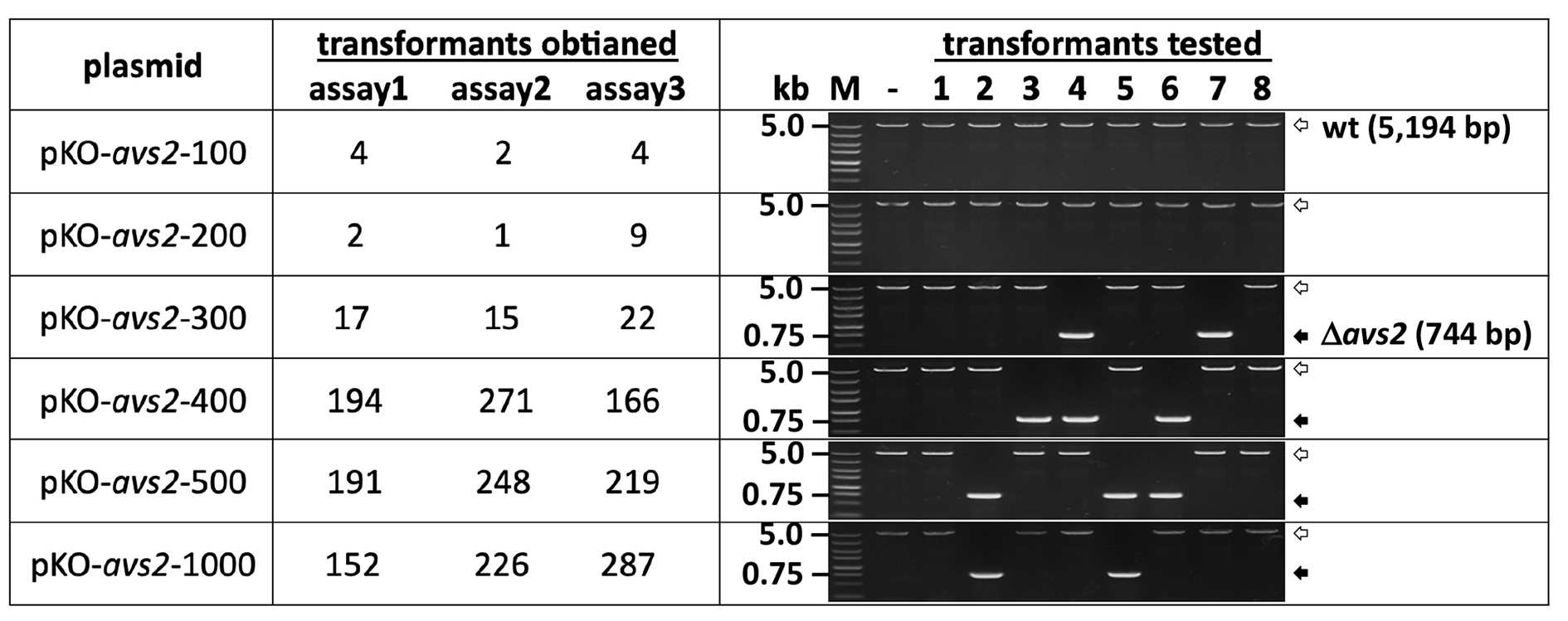


**Fig S1. Examination of the sufficiency of donor sizes for facilitating genome editing.**  Number of transformants obtained from three independent transformation assays with pKO-*avs2* derivatives are given. Sizes of homologous arms in each plasmid are shown, *e.g.* 100 bp UF + 100 bp DF in pKO-*avs2*-100. Randomly selected strains were analyzed by colony PCR and the predicted sizes of replicons in wild-type (wt) and the intended Δ*avs2* mutants are indicated with unfilled and filled arrows, respectively. -, amplicon of a pAE1 transformant; M, DNA size marker.

**
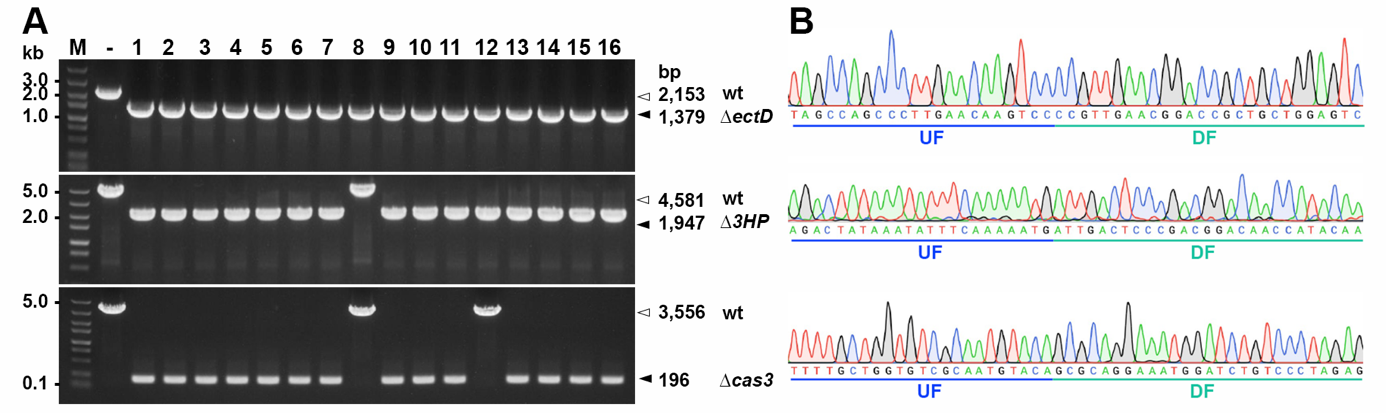
**

**Fig S2. Efficient knockout of genes of difference sizes using the optimized genome editing platform in *A. faecalis*.**

**(A)** Colony PCR screening of deletion mutants of *ectD*, *3PH* operon, and *cas3*. Predicted sizes of PCR products in wild-type (wt) and the intended deletion mutans (Δ*ectD*, Δ*3PH* or Δ*cas3*) are indicated with unfilled and filled arrowheads, respectively. -, PCR amplification using genomic DNA of *A. faecalis* J481 as a DNA template. M, DNA size marker. **(B)** Representative chromatographs of Sanger sequencing results for the corresponding deletions. Up-flanking (UF) and down-flanking (DF) sequences of each target site are underlined in blue and green, respectively.

**Table S1**. *Alcaligenes faecalis* strains and plasmids used or constructed in this work.

| **Strain/Plasmid** | **Genotype and features** | **Source** |
| --- | --- | --- |
| **Strains** |  |  |
| J481 | An *Alcaligenes faecalis* wild-type strain | CP032521 |
| Δ*mrr* | A J481 derivative with the *mrr* gene disrupted | This work |
| Δ*avs2* | A derivative of J481 or Δ*mrr* with the *avs2* gene disrupted | This work |
| cQatA-HT | A derivative Δ*mrr* with the *qatA* gene chromosomally His-tagged | This work |
| Δ30k | A Δ*mrr* derivative with a 29,554-bp genomic deletion | This work |
| Δ47k | A Δ30k derivative with a 17,343-bp genomic deletion | This work |
| **Plasmids** |  |  |
| pAE1 | An *Alcaligenes faecalis*-*Escherichia coli* shuttle vector containing the pBBR1 replicon and the ColE1 origin of replication; amp^r^ | This work |
| pInt-A1S1 | An artificial interference plasmid; pAE1 containing a protospacer sequence of CRISPR Array1 Spacer1 preceded by a 5’-CCC-3’ PAM | This work |
| pRef-A1S1 | Reference plasmid of pInt-A1S1; the PAM was replaced with the last 3 nt of repeat sequence (5’-AAA-3’) | This work |
| pInt-A2S6 | An artificial interference plasmid; pAE1 containing a protospacer sequence of CRISPR Array2 Spacer6 preceded by a 5’-CCC-3’ PAM | This work |
| pRef-A2S6 | Reference plasmid of pInt-A2S6; the PAM was replaced with the last 3 nt of repeat sequence (5’-AAA-3’) | This work |
| pELB | pAE1 expressing the *E. coli* lambda phage *B* gene | This work |
| pSpy | A broad host range cloning vector; kan^r^ | PP457283 |
| pAvs2 | pSpy carrying the *A. faecalis* J481 *avs2* gene | This work |
| pQatA-HT | pSpy carrying the the *A. faecalis* cQatA-HT *qatA* gene | This work |
| pVGE | pAE1 carrying a DNA fragment of two tandem copies of CRISPR repeats; a basal vector for construction of genome editing plasmids | This work |
| pPheSv | pVGE derivative expressing a mutant of PheS (PheSv marker) | This work |
| pKO-*avs2*  (pKO-*avs2*-500) | pVGE derivative containing a CRISPR locus with a spacer matching a protospacer in *avs2*, and a donor of recombination arms (500 bp UF + 500 bp DF) homologous to the sequences flanking *avs2* | This work |
| pKO-*avs2*-100 | pVGE derivative containing a CRISPR locus with a spacer matching a protospacer in *avs2*, and a donor of recombination arms (100 bp UF + 100 bp DF) homologous to the sequences flanking *avs2* | This work |
| pKO-*avs2*-200 | pVGE derivative containing a CRISPR locus with a spacer matching a protospacer in *avs2*, and a donor of recombination arms (200 bp UF + 200 bp DF) homologous to the sequences flanking *avs2* | This work |
| pKO-*avs2*-300 | pVGE derivative containing a CRISPR locus with a spacer matching a protospacer in *avs2*, and a donor of recombination arms (300 bp UF + 300 bp DF) homologous to the sequences flanking *avs2* | This work |
| pKO-*avs2*-400 | pVGE derivative containing a CRISPR locus with a spacer matching a protospacer in *avs2*, and a donor of recombination arms (400 bp UF + 400 bp DF) homologous to the sequences flanking *avs2* | This work |
| pKO-*avs2*-1000 | pVGE derivative containing a CRISPR locus with a spacer matching a protospacer in *avs2*, and a donor of recombination arms (1000 bp UF + 1000 bp DF) homologous to the sequences flanking *avs2* | This work |
| pHT-*qatA* | pVGE derivative containing a CRISPR locus with a spacer matching a protospacer in *qatA*, and a donor carrying a G4S-6xHis coding sequence immediately upstream of the stop codon of qatA | This work |
| pDel-30k | pVGE derivative containing a CRISPR locus with a spacer matching a protospacer in *D6I95_00425*, and a donor of recombination arms for deletion of a 29,554-bp genomic region | This work |
| pDel-30kv | pDel-30k carrying the PheSv marker | This work |
| pDel-17k | pVGE derivative containing a CRISPR locus with a spacer matching a protospacer in *D6I95_14430*, and a donor of recombination arms for deletion of a 17,343-bp genomic region | This work |

**Table S2**. Oligonucleotides used in this work. Restriction sites or protruding nucleotides in bold and overlapped sequences for SOE-PCR and for recombination during one-step cloning underlined.

| **Oligonucleotide** | **Sequence (**5’-3’**)** |
| --- | --- |
| pBBR1(pUC)-F | CCCCGTAGAAAAGATCAAAGGATCTTCCTACCGGCGCGGCAGCGTGA |
| pBBR1(pUC)-R | AAGGCCGCGTTGCTGGCGTTGCGGCCACCGGCTGGCTCGC |
| pUC(pBBR1)-F | GCGAGCCAGCCGGTGGCCGCAACGCCAGCAACGCGGCCT |
| pUC(pBBR1)-R | TCACGCTGCCGCGCCGGTAGGAAGATCCTTTGATCTTTTCTACGGGGTC |
| PheS-F | CGCC**AAGCTT**GCGGTATCTGACTGTATAGT |
| T259A-R | CGATGGCTCGGCAAAGGGGAAGAAAGAAGG |
| T259A-F | TCCCCTTTGCCGAGCCATCGGCCGA |
| A306G-R | GGAACCAAAGCCGAAACCGATGTAACGCTCTG |
| A306G-F | ATCGGTTTCGGCTTTGGTTCCGGACTGGAGCG |
| PheS-R | TAGA**GTCGAC**CGGCGTATACCGGAACCGAC |
| Avs2-F | GAGA**GAGCTC**GACACAGCGGCTGTGATTGA |
| Avs2-R | TTAA**TCTAGA**AACAGAAATTGATATTGCCG |
| CCC-A1S1-F | **AGCT**CCCGTACAGACGCGCCAACAGGCACGTCAACAATA |
| CCC-A1S1-R | **GATC**TATTGTTGACGTGCCTGTTGGCGCGTCTGTACGGG |
| AAA-A1S1-F | **AGCT**AAAGTACAGACGCGCCAACAGGCACGTCAACAATA |
| AAA-A1S1-R | **GATC**TATTGTTGACGTGCCTGTTGGCGCGTCTGTACTTT |
| CCC-A2S6-F | **AGCT**CCCATGCCGTGGTGTCTGTTGGGCTGGATGGCACTG |
| CCC-A2S6-R | **GATC**CAGTGCCATCCAGCCCAACAGACACCACGGCATGGG |
| AAA-A2S6-F | **AGCT**AAAATGCCGTGGTGTCTGTTGGGCTGGATGGCACTG |
| AAA-A2S6-R | **GATC**CAGTGCCATCCAGCCCAACAGACACCACGGCATTTT |
| Sp-*mrr*-F | **GAAA**TCACTGCTGGACAAGCTGCTTCTAACGACTTC |
| Sp-*mrr*-R | **GAAC**GAAGTCGTTAGAAGCAGCTTGTCCAGCAGTGA |
| Sp-*avs2*-F | **GAAA**CAAAGTTTGATCTGAAGCTGGTGCAACGTTAT |
| Sp-*avs2*-R | **GAAC**ATAACGTTGCACCAGCTTCAGATCAAACTTTG |
| Sp-30k-F | **GAAA**ATCGCTACAAGGAGCTACGGGTTTTGCAGCAG |
| Sp-30k-R | **GAAC**CTGCTGCAAAACCCGTAGCTCCTTGTAGCGAT |
| Sp-17k-F | **GAAA**AGCGCGCCGTGGAACACGGCTGGTCACGCGCC |
| Sp-17k-R | **GAAC**GGCGCGTGACCAGCCGTGTTCCACGGCGCGCT |
| Sp-*qat*-F | **GAAA**CATTATCGGACTCCCTTCGGTGGTTTCAGTGC |
| Sp-*qat*-R | **GAAC**GCACTGAAACCACCGAAGGGAGTCCGATAATG |
| *mrr*-UF-F | TGCCTGCAGGTCGACCAAAGCATCGAATGTACCCG |
| *mrr*-UF-R | TTTCTTCATCGTTGCATCCGTGGCATTGGT |
| *mrr*-DF-F | CGGATGCAACGATGAAGAAACAATGGCGTA |
| *mrr*-DF-R | GTCCCCCGGGGATCCGATGGCTTGCTGATCTCCGG |
| *mrr*_chkF | CGTAGACGGAAAGGTAACAA |
| *mrr*_chkR | GGCAGTGACTGGCAACCCTA |
| *avs2*-UF-F | TGCCTGCAGGTCGACCTGGGCCTACGCGTGCTGCG |
| *avs2*-UF-R | AGACGAAGAGGCGCGGATTGATGTCCAATC |
| *avs2*-DF-F | CAATCCGCGCCTCTTCGTCTGGGATGGTGT |
| *avs2*-DF-R | GTCCCCCGGGGATCCGACAATGCTATTGACGAGTT |
| *avs2*_chkF | TTCGGCGTTGATCCCTGACG |
| *avs2*_chkR | TGGACCTAAAACAGAAATTG |
| *qatA*_chkF | CGGGTGTCGACTTGAGACCC |
| His-R | ATGGTGATGGTGATGGTGGG |
| *qatA*_chkR | TTCATCGACGGTGCCCGCTC |
| 30k-UF-F | TGCCTGCAGGTCGACGACAGCTTTGGAGAACAGAT |
| 30k-UF-R | GAGGTGGCAACAGTATCGTAGCCTGTCGTT |
| 30k-DF-F | TACGATACTGTTGCCACCTCATGAAGTTCT |
| 30k-DF-R | GTCCCCCGGGGATCCATCGGTCACAATGTGGACTA |
| 30k_chkF | CCGTTGAATCAAGGCAATCA |
| 30k_chkR | GGCAGCTCTCCGAGCAGG |
| 17k-UF-F | TGCCTGCAGGTCGACGGCATGCCACGCCAAGCCTT |
| 17k-UF-R | TCGCTCATGCAAGCCAACGATGCCGCATTG |
| 17k-DF-F | TCGTTGGCTTGCATGAGCGAGGCCGGAGTA |
| 17k-DF-R | GTCCCCCGGGGATCCGGACATCCTCTCGGTTACCA |
| 17k_chkF | GGCTGTCTGGATCAGCCGTT |
| 17k_chkR | CCTTTGCGAGAATCGCTGGA |
| Syn1 | AATT**CCCGGG**GGACCGGAGCAGCCGCTTGCAGCGGCTGGATGTTTCAATGCTTACGGGCTAAGTGCGCAGGCTAGCATTCCCTGGTTTTGACCCTAATTTTCAGTGGAAATAATTTTTCTTTTAAATCAATGGATTATGTTTTTGTCTAAAAAAAGGGTTTAATAGGTGAAAAAGTCTGAAAGTGCTGAGTATTCAGTGTTTTCAGGCTTTTTGCCTCTAGTTCGCTGCCGCATAGGCAGCTTAGAAAGGAGACCGAGGTCTCAGTTCGCTGCCGCATAGGCAGCTTAGAAATTCAACCTTAAGGGCGTGAGCGTCACCATGGCGTTTGCTGTGTGCAATCTTCGCCTCTTATTGAGAATCGGCAGCAAAATCCATGTTCATTGAGCATGCCCGATTCAGCCTATATTGGTT**GAGCTC**GTAC |
| Syn2 | TGCCTGCAGGTCGACCGGGTGTCGACTTGAGACCCTATCTCTTTGTCACCAAGGATAAGAAGGACTACTTCGGGCCGGTTTCCGTGTTGGGACATTTGGCGGGCGTGGTGGATAAGCTGTTCGGCGGCAAGATGACCGTACAAGGCTATGAGGCAGAGTTGAAGCAATTGGCTCAGCCTGAAGCTGACAAGGTATTTGAGGCCGTGCGTAGTCGAATCACGAGTACCGGCGCTTTTGATACCAAGCCGGACGGCATTGACGGGCTTATCGTGCTCGTGAAGGCTCAACCGAGCCTGCAAGAACCTTTGCTGGATTTTCTGGAAGCACTGCCTCGAGAAAAATGCGGAGCCTGGGTAGTCGGGGGCTGGCAAGGAGCCGTCAAGGATACAGAGTGCAGTGCTCGTTTACTCAAGCTATTGGGTGAATGGAGCAAGGTAACGAAGAATCCTTTCTTGAAGGCCGCAGCAGAGAACGCACTGAAACCACCGAAGGGAGTCCGAGGAGGTGGGGGATCCCACCATCACCATCACCATTAATGGGGACGTCAACAGCATATGGCGGCCCGGGTGGAGACACGCCATTGGTTCCCTCGTGGTTGGGGGATCCACCAGCATCACCGCCAGCGAATCCTGATGGAGCCCCAGACGGAACGCCACCACCTGATGCAGTCGACCCACCCTCGCCTCCGGAGAAGCCCCCAATACCAAAGGTCGCGGATCCGCAGCGCTTTTCAGGCGCACGCAACAACCTTACGAGATTCGCAGGATCTGGCGGTAGTGATCGAACCAACCTTGGCCGAGCCATCTCGCGGTATGTCTCCACATCCTCTGGCGGCGCTCGCCAAGCAGCGCAACGGATGGGCACATCACGCAGTGCCGGCGCAAGACTGCTTGGATTCCTTGCCGATGCGAATGCGCGAGGGATGCGCGAAGCGCTGCGTGAGTTCAATCTTGACTCCATGGCGGGACGACCCGTCTCGGAAGTATTCATTGCGCTGGCCGATCACATCTGTCCGGGAGCGGGCACCGTCGATGAAGTCCCCCGGGGATCC |
| Syn3 | CAG**GTCGAC**CAGGTGCTGAAAGCGAGGCTTTTTGGCCTCTGTCGTTTCCTTTCTCTGTTTTTGTCCGTGGAATGAACAGGTTATCACGGCGGTGGCAGCGGATTTGGAGGGCAGTTGCGGTCGTGGAACCCACCGAGTGAAAGTGTGGATGCAGCCCTGTTGCCCAACTTTACCCGTGGCAATGCCCGCGCAGACGATCTGGTACGCAATAACGGCTATGCCGCCAACGCCATCCAGCTGCATCAGGATCATATCGTCGGGTCTTTTTTCCGGCTCAGTCATCGCCCAAGCTGGCGCTATCTGGGCATCGGGGAGGAAGAAGCCCGTGCCTTTTCCCGCGAGGTTGAAGCGGCATGGAAAGAGTTTGCCGAGGATGACTGCTGCTGCATTGACGTTGAGCGAAAACGCACGTTTACCATGATGATTCGGGAAGGTGTGGCCATGCACGCCTTTAACGGTGAACTGTTCGTTCAGGCCACCTGGGATACCAGTTCGTCGCGGCTTTTCCGGACACAGTTCCGGATGGTCAGCCCGAAGCGCATCAGCAACCCGAACAATACCGGCGACAGCCGGAACTGCCGTGCCGGTGTGCAGATTAATGACAGCGGTGCGGCGCTGGGATATTACGTCAGCGAGGACGGGTATCCTGGCTGGATGCCGCAGAAATGGACATGGATACCCCGTGAGTTACCCGGCGGGCGCGCCTCGTTCATTCACGTTTTTGAACCCGTGGAGGACGGGCAGACTCGCGGTGCAAATGTGTTTTACAGCGTGATGGAGCAGATGAAGATGCTCGACACGCTGCAGAACACGCAGCTGCAGAGCGCCATTGTGAAGGCGATGTATGCCGCCACCATTGAGAGTGAGCTGGATACGCAGTCAGCGATGGATTTTATTCTGGGCGCGAACAGTCAGGAGCAGCGGGAAAGGCTGACCGGCTGGATTGGTGAAATTGCCGCGTATTACGCCGCAGCGCCGGTCCGGCTGGGAGGCGCAAAAGTACCGCACCTGATGCCGGGTGACTCACTGAACCTGCAGACGGCTCAGGATACGGATAACGGCTACTCCGTGTTTGAGCAGTCACTGCTGCGGTATATCGCTGCCGGGCTGGGTGTCTCGTATGAGCAGCTTTCCCGGAATTACGCCCAGATGAGCTACTCCACGGCACGGGCCAGTGCGAACGAGTCGTGGGCGTACTTTATGGGGCGGCGAAAATTCGTCGCATCCCGTCAGGCGAGCCAGATGTTTCTGTGCTGGCTGGAAGAGGCCATCGTTCGCCGCGTGGTGACGTTACCTTCAAAAGCGCGCTTCAGTTTTCAGGAAGCCCGCAGTGCCTGGGGGAACTGCGACTGGATAGGCTCCGGTCGTATGGCCATCGATGGTCTGAAAGAAGTTCAGGAAGCGGTGATGCTGATAGAAGCCGGACTGAGTACCTACGAGAAAGAGTGCGCAAAACGCGGTGACGACTATCAGGAAATTTTTGCCCAGCAGGTCCGTGAAACGATGGAGCGCCGTGCAGCCGGTCTTAAACCGCCCGCCTGGGCGGCTGCAGCATTTGAATCCGGGCTGCGACAATCAACAGAGGAGGAGAAGAGTGACAGCAGAGCTGCGTAATCTCCCGCATATTGCCAGCATGGCCTTTAATGAGCCGCTGATGCTTGAACCCGCCTATGCGCGGGTTTTCTTTTGTGCGCTTGC**GAATTC**ACT |

**Table S3**. Complete sequences of the *A. faecalis*-*E. coli* shuttle vector pAE1 and the basal vector pVGE.

| **Plasmid** | **Sequence** |
| --- | --- |
| pAE1 | gacgaaagggcctcgtgatacgcctatttttataggttaatgtcatgataataatggtttcttagacgtcaggtggcacttttcggggaaatgtgcgcggaacccctatttgtttatttttctaaatacattcaaatatgtatccgctcatgagacaataaccctgataaatgcttcaataatattgaaaaaggaagagtatgagtattcaacatttccgtgtcgcccttattcccttttttgcggcattttgccttcctgtttttgctcacccagaaacgctggtgaaagtaaaagatgctgaagatcagttgggtgcacgagtgggttacatcgaactggatctcaacagcggtaagatccttgagagttttcgccccgaagaacgttttccaatgatgagcacttttaaagttctgctatgtggcgcggtattatcccgtattgacgccgggcaagagcaactcggtcgccgcatacactattctcagaatgacttggttgagtactcaccagtcacagaaaagcatcttacggatggcatgacagtaagagaattatgcagtgctgccataaccatgagtgataacactgcggccaacttacttctgacaacgatcggaggaccgaaggagctaaccgcttttttgcacaacatgggggatcatgtaactcgccttgatcgttgggaaccggagctgaatgaagccataccaaacgacgagcgtgacaccacgatgcctgtagcaatggcaacaacgttgcgcaaactattaactggcgaactacttactctagcttcccggcaacaattaatagactggatggaggcggataaagttgcaggaccacttctgcgctcggcccttccggctggctggtttattgctgataaatctggagccggtgagcgtgggtctcgcggtatcattgcagcactggggccagatggtaagccctcccgtatcgtagttatctacacgacggggagtcaggcaactatggatgaacgaaatagacagatcgctgagataggtgcctcactgattaagcattggtaactgtcagaccaagtttactcatatatactttagattgatttaaaacttcatttttaatttaaaaggatctaggtgaagatcctttttgataatctcatgaccaaaatcccttaacgtgagttttcgttccactgagcgtcagaccccgtagaaaagatcaaaggatcttcctaccggcgcggcagcgtgacccgtgtcggcggctccaacggctcgccatcgtccagaaaacacggctcatcgggcatcggcaggcgctgctgcccgcgccgttcccattcctccgtttcggtcaaggctggcaggtctggttccatgcccggaatgccgggctggctgggcggctcctcgccggggccggtcggtagttgctgctcgcccggatacagggtcgggatgcggcgcaggtcgccatgccccaacagcgattcgtcctggtcgtcgtgatcaaccaccacggcggcactgaacaccgacaggcgcaactggtcgcggggctggccccacgccacgcggtcattgaccacgtaggccgacacggtgccggggccgttgagcttcacgacggagatccagcgctcggccaccaagtccttgactgcgtattggaccgtccgcaaagaacgtccgatgagcttggaaagtgtcttctggctgaccaccacggcgttctggtggcccatctgcgccacgaggtgatgcagcagcattgccgccgtgggtttcctcgcaataagcccggcccacgcctcatgcgctttgcgttccgtttgcacccagtgaccgggcttgttcttggcttgaatgccgatttctctggactgcgtggccatgcttatctccatgcggtaggggtgccgcacggttgcggcaccatgcgcaatcagctgcaacttttcggcagcgcgacaacaattatgcgttgcgtaaaagtggcagtcaattacagattttctttaacctacgcaatgagctattgcggggggtgccgcaatgagctgttgcgtaccccccttttttaagttgttgatttttaagtctttcgcatttcgccctatatctagttctttggtgcccaaagaagggcacccctgcggggttcccccacgccttcggcgcggctccccctccggcaaaaagtggcccctccggggcttgttgatcgactgcgcggccttcggccttgcccaaggtggcgctgcccccttggaacccccgcactcgccgccgtgaggctcggggggcaggcgggcgggcttcgcccttcgactgcccccactcgcataggcttgggtcgttccaggcgcgtcaaggccaagccgctgcgcggtcgctgcgcgagccttgacccgccttccacttggtgtccaaccggcaagcgaagcgcgcaggccgcaggccggaggcttttccccagagaaaattaaaaaaattgatggggcaaggccgcaggccgcgcagttggagccggtgggtatgtggtcgaaggctgggtagccggtgggcaatccctgtggtcaagctcgtgggcaggcgcagcctgtccatcagcttgtccagcagggttgtccacgggccgagcgaagcgagccagccggtggccgcaacgccagcaacgcggcctttttacggttcctggccttttgctggccttttgctcacatgttctttcctgcgttatcccctgattctgtggataaccgtattaccgcctttgagtgagctgataccgctcgccgcagccgaacgaccgagcgcagcgagtcagtgagcgaggaagcggaagagcgcccaatacgcaaaccgcctctccccgcgcgttggccgattcattaatgcagctggcacgacaggtttcccgactggaaagcgggcagtgagcgcaacgcaattaatgtgagttagctcactcattaggcaccccaggctttacactttatgcttccggctcgtatgttgtgtggaattgtgagcggataacaatttcacacaggaaacagctatgaccatgattacgccaagcttgcatgcctgcaggtcgactctagaggatccccgggtaccgagctcgaattcactggccgtcgttttacaacgtcgtgactgggaaaaccctggcgttacccaacttaatcgccttgcagcacatccccctttcgccagctggcgtaatagcgaagaggcccgcaccgatcgcccttcccaacagttgcgcagcctgaatggcgaatggcgcctgatgcggtattttctccttacgcatctgtgcggtatttcacaccgcatatggtgcactctcagtacaatctgctctgatgccgcatagttaagccagccccgacacccgccaacacccgctgacgcgccctgacgggcttgtctgctcccggcatccgcttacagacaagctgtgaccgtctccgggagctgcatgtgtcagaggttttcaccgtcatcaccgaaacgcgcga |
| pVGE | gcgcccaatacgcaaaccgcctctccccgcgcgttggccgattcattaatgcagctggcacgacaggtttcccgactggaaagcgggcagtgagcgcaacgcaattaatgtgagttagctcactcattaggcaccccaggctttacactttatgcttccggctcgtatgttgtgtggaattgtgagcggataacaatttcacacaggaaacagctatgaccatgattacgccaagcttgcatgcctgcaggtcgactctagaggatccccgggggaccggagcagccgcttgcagcggctggatgtttcaatgcttacgggctaagtgcgcaggctagcattccctggttttgaccctaattttcagtggaaataatttttcttttaaatcaatggattatgtttttgtctaaaaaaagggtttaataggtgaaaaagtctgaaagtgctgagtattcagtgttttcaggctttttgcctctagttcgctgccgcataggcagcttagaaaGGAGACCGAGGTCTCAgttcgctgccgcataggcagcttagaaattcaaccttaagggcgtgagcgtcaccatggcgtttgctgtgtgcaatcttcgcctcttattgagaatcggcagcaaaatccatgttcattgagcatgcccgattcagcctatattggttgagctcgaattcactggccgtcgttttacaacgtcgtgactgggaaaaccctggcgttacccaacttaatcgccttgcagcacatccccctttcgccagctggcgtaatagcgaagaggcccgcaccgatcgcccttcccaacagttgcgcagcctgaatggcgaatggcgcctgatgcggtattttctccttacgcatctgtgcggtatttcacaccgcatatggtgcactctcagtacaatctgctctgatgccgcatagttaagccagccccgacacccgccaacacccgctgacgcgccctgacgggcttgtctgctcccggcatccgcttacagacaagctgtgaccgtctccgggagctgcatgtgtcagaggttttcaccgtcatcaccgaaacgcgcgagacgaaagggcctcgtgatacgcctatttttataggttaatgtcatgataataatggtttcttagacgtcaggtggcacttttcggggaaatgtgcgcggaacccctatttgtttatttttctaaatacattcaaatatgtatccgctcatgagacaataaccctgataaatgcttcaataatattgaaaaaggaagagtatgagtattcaacatttccgtgtcgcccttattcccttttttgcggcattttgccttcctgtttttgctcacccagaaacgctggtgaaagtaaaagatgctgaagatcagttgggtgcacgagtgggttacatcgaactggatctcaacagcggtaagatccttgagagttttcgccccgaagaacgttttccaatgatgagcacttttaaagttctgctatgtggcgcggtattatcccgtattgacgccgggcaagagcaactcggtcgccgcatacactattctcagaatgacttggttgagtactcaccagtcacagaaaagcatcttacggatggcatgacagtaagagaattatgcagtgctgccataaccatgagtgataacactgcggccaacttacttctgacaacgatcggaggaccgaaggagctaaccgcttttttgcacaacatgggggatcatgtaactcgccttgatcgttgggaaccggagctgaatgaagccataccaaacgacgagcgtgacaccacgatgcctgtagcaatggcaacaacgttgcgcaaactattaactggcgaactacttactctagcttcccggcaacaattaatagactggatggaggcggataaagttgcaggaccacttctgcgctcggcccttccggctggctggtttattgctgataaatctggagccggtgagcgtggAtctcgcggtatcattgcagcactggggccagatggtaagccctcccgtatcgtagttatctacacgacggggagtcaggcaactatggatgaacgaaatagacagatcgctgagataggtgcctcactgattaagcattggtaactgtcagaccaagtttactcatatatactttagattgatttaaaacttcatttttaatttaaaaggatctaggtgaagatcctttttgataatctcatgaccaaaatcccttaacgtgagttttcgttccactgagcgtcagaccccgtagaaaagatcaaaggatcttcctaccggcgcggcagcgtgacccgtgtcggcggctccaacggctcgccatcgtccagaaaacacggctcatcgggcatcggcaggcgctgctgcccgcgccgttcccattcctccgtttcggtcaaggctggcaggtctggttccatgcccggaatgccgggctggctgggcggctcctcgccggggccggtcggtagttgctgctcgcccggatacagggtcgggatgcggcgcaggtcgccatgccccaacagcgattcgtcctggtcgtcgtgatcaaccaccacggcggcactgaacaccgacaggcgcaactggtcgcggggctggccccacgccacgcggtcattgaccacgtaggccgacacggtgccggggccgttgagcttcacgacggagatccagcgctcggccaccaagtccttgactgcgtattggaccgtccgcaaagaacgtccgatgagcttggaaagtgtTttctggctgaccaccacggcgttctggtggcccatctgcgccacgaggtgatgcagcagcattgccgccgtgggtttcctcgcaataagcccggcccacgcctcatgcgctttgcgttccgtttgcacccagtgaccgggcttgttcttggcttgaatgccgatttctctggactgcgtggccatgcttatctccatgcggtaggggtgccgcacggttgcggcaccatgcgcaatcagctgcaacttttcggcagcgcgacaacaattatgcgttgcgtaaaagtggcagtcaattacagattttctttaacctacgcaatgagctattgcggggggtgccgcaatgagctgttgcgtaccccccttttttaagttgttgatttttaagtctttcgcatttcgccctatatctagttctttggtgcccaaagaagggcacccctgcggggttcccccacgccttcggcgcggctccccctccggcaaaaagtggcccctccggggcttgttgatcgactgcgcggccttcggccttgcccaaggtggcgctgcccccttggaacccccgcactcgccgccgtgaggctcggggggcaggcgggcgggcttcgcccttcgactgcccccactcgcataggcttgggtcgttccaggcgcgtcaaggccaagccgctgcgcggtcgctgcgcgagccttgacccgccttccacttggtgtccaaccggcaagcgaagcgcgcaggccgcaggccggaggcttttccccagagaaaattaaaaaaattgatggggcaaggccgcaggccgcgcagttggagccggtgggtatgtggtcgaaggctgggtagccggtgggcaatccctgtggtcaagctcgtgggcaggcgcagcctgtccatcagcttgtccagcagggttgtccacgggccgagcgaagcgagccagccggtggccgcaacgccagcaacgcggcctttttacggttcctggccttttgctggccttttgctcacatgttctttcctgcgttatcccctgattctgtggataaccgtattaccgcctttgagtgagctgataccgctcgccgcagccgaacgaccgagcgcagcgagtcagtgagcgaggaagcggaaga |
